# Ischemia–Reperfusion Injury in Lung Transplantation

**DOI:** 10.3390/cells10061333

**Published:** 2021-05-28

**Authors:** Toyofumi Fengshi Chen-Yoshikawa

**Affiliations:** Department of Thoracic Surgery, Graduate School of Medicine, Nagoya University, Nagoya 466-8550, Japan; tyoshikawa@med.nagoya-u.ac.jp; Tel.: +81-52-744-2375; Fax: +81-52-744-2382

**Keywords:** endothelial protection, ex vivo lung perfusion, ischemia–reperfusion injury, lung transplantation, primary graft dysfunction

## Abstract

Lung transplantation has been established worldwide as the last treatment for end-stage respiratory failure. However, ischemia–reperfusion injury (IRI) inevitably occurs after lung transplantation. The most severe form of IRI leads to primary graft failure, which is an important cause of morbidity and mortality after lung transplantation. IRI may also induce rejection, which is the main cause of mortality in recipients. Despite advances in donor management and graft preservation, most donor grafts are still unsuitable for transplantation. Although the pulmonary endothelium is the primary target site of IRI, the pathophysiology of lung IRI remains incompletely understood. It is essential to understand the mechanism of pulmonary IRI to improve the outcomes of lung transplantation. Therefore, we reviewed the state-of-the-art in the management of pulmonary IRI after lung transplantation. Recently, the ex vivo lung perfusion (EVLP) system has been clinically introduced worldwide. Various promising therapeutic strategies for the protection of the endothelium against IRI, including EVLP, inhalation therapy with therapeutic gases and substances, fibrinolytic treatment, and mesenchymal stromal cell therapy, are awaiting clinical application. We herein review the latest advances in the field of pulmonary IRI in lung transplantation.

## 1. Introduction

Lung transplantation is currently the only viable treatment for end-stage respiratory diseases worldwide [1]. However, chronic donor shortage remains a grave issue in lung transplantation and >80% of donor graft lungs are unsuitable for transplantation for various reasons [1,2]. The process of ischemia followed by reperfusion occurs in various medical situations, including lung transplantation. This process can lead to devastating consequences in some patients, referred to as ischemia–reperfusion injury (IRI). Pulmonary IRI after lung transplantation is the main reason for primary graft dysfunction (PGD), which is a major cause of mortality and morbidity in the postoperative period. Major efforts have been undertaken to improve the understanding of IRI in order to reduce the incidence of PGD and even treat fatal PGD [3,4].

In lung transplantation, the donor lungs are procured from the donor and transferred to the recipient. Until reperfusion is initiated, the donor lungs are preserved in an ischemic state. Organ ischemia begins with an imbalance between the metabolic supply and demand and ends with tissue hypoxia, eventually leading to cellular damage or death. Restoration of adequate organ perfusion is the ultimate treatment; however, the re-establishment of perfusion in the ischemic lung also involves the activation of inflammatory cells/mediators and reactive oxygen species (ROS) that promote further injury, resulting in increased apoptosis and contributing to pulmonary dysfunction [5]. The pulmonary endothelium is the primary target site of IRI, which potentially results in severe pulmonary dysfunction accompanied by the rapid development of pulmonary edema due to the increased endothelial permeability, increased pulmonary vascular resistance, and decreased airway compliance.

Patients with IRI require prolonged mechanical ventilation with prolonged hospital stays. Furthermore, IRI is a risk factor for late graft failure, such as chronic lung allograft dysfunction [6]. Therefore, it is essential to understand the mechanism of lung IRI for its prevention and treatment. In this review, we discuss the underlying mechanisms of pulmonary IRI related to lung transplantation.

## 2. Clinical Dilemma in Lung Transplantation

The number of lung transplantation procedures has increased since the first successful operation by the Toronto Group in 1983 [7]. To date, >70,000 lung transplantations have been performed worldwide. The current 5-year survival rate after lung transplantation is approximately 55% according to the International Society of Heart and Lung Transplantation registry data [8], making lung transplantation a satisfactory therapeutic option for end-stage respiratory failure in the absence of other options. However, lung transplantation still has various problems, including PGD, rejection, infection, surgical complications, malignancy, and chronic lung allograft dysfunction [9]. Among them, PGD represents one of the most frequent causes of early mortality [8]. In general, PGD is caused by IRI, which is defined as an injury due to the interruption (ischemia) and reopening (reperfusion) of blood flow to the organ. Therefore, an increased understanding of the mechanism of IRI and the strategies to reduce its occurrence can improve the function of the transplanted organ and the outcome of lung transplantation.

## 3. Ischemia–Reperfusion Injury

In lung transplantation, organ ischemia followed by reperfusion is unavoidable and commonly leads to acute, sterile inflammation after transplantation, which is called IRI. During the time between the procurement of donor lungs and their reperfusion after implantation, the organs are exposed to ischemia (i.e., cessation of blood flow) and severe hypoxia. Ischemia can rapidly lead to a cascade of pathologic changes in the cells, including decreased activity of life-sustaining systems, resulting in energy depletion [10]. The damage caused by ischemia ultimately results in cell death accompanied by the release of damage-associated molecules [11]. These molecules can bind to their corresponding receptors, eventually leading to the upregulation of cytokine production, with devastating inflammatory reactions upon reperfusion [12]. During ischemia, energy depletion processes, such as adenosine triphosphate (ATP) metabolism, result in the accumulation of hypoxanthine, which also induces oxidative stress upon reperfusion [3]. Furthermore, the lack of shear stress on the vascular endothelium results in a sudden increase in ROS generation [13]. Several studies have shown that ischemia in lung transplantation can result in relevant vascular endothelial structural changes, such as increased vascular permeability and impaired pulmonary vasodilatation [14,15,16]. Reperfusion generally exacerbates these ischemia-related responses by inducing leukocyte sequestration and activation in the pulmonary circulation, the activation of the complement system, and the release of inflammatory mediators. IRI is characterized by the rapid accumulation of ROS soon after reperfusion, with increased activities of ROS-generating enzymes [13]. IRI is also associated with a cascade of proinflammatory changes, including the upregulation of cytokines after reperfusion [17].

### 3.1. Changes in Ischemic Storage

Hypothermia is considered an essential component of the storage of donor lungs. Because hypothermia decreases the metabolic rate, it also results in reduced biochemical reactions and decreased rate of degradation of the cellular components necessary for the viability of the donor lungs [3,4]. However, in hypothermic organ preservation, multiple donor-associated changes can alter the condition of the donor organ. For example, the Na-K adenosine triphosphatase activity decreases, and Na^+^ and Cl^−^ flow with water from the extracellular space into the intracellular space, which can result in cellular edema. Dysregulated calcium homeostasis induces intracellular calcium overload, resulting in the disruption of many intracellular processes and causing severe cellular damage [3,4,18]. In organ preservation, saccharides are an essential energy source during ischemia and are also considered to prevent cellular edema by acting as an impermeant [4,19]. Several types of saccharides, such as monosaccharide (glucose), disaccharides (trehalose), and trisaccharides (raffinose), have been used in lung preservation; however, the best saccharide for use in organ preservation solutions has not yet been identified [19].

Interestingly, during ischemic storage, ROS are also produced in various cells, leading to robust oxidative stress responses [20]. One mechanism is derived from the accumulation of hypoxanthine, which is the main ATP degradation product in ischemic tissues and the upregulated xanthine oxidase activity [3]. Xanthine oxidase catalyzes the oxidation of hypoxanthine to xanthine and that of xanthine to uric acid, and both steps generate hydrogen peroxide. The conversion of xanthine oxidase from its precursor enzyme is augmented by hypoxia. Another mechanism depends on nicotinamide adenine dinucleotide phosphate (NADPH) oxidase, which is the only known enzyme whose primary function is ROS generation and is widely expressed in pulmonary cells including vascular, immune, and alveolar cells [3,21,22].

Shear stress-related mechanosignaling is also one of the key factors in pulmonary IRI. Cessation of blood flow represents a physical event that is sensed by the pulmonary endothelium, leading to a signaling cascade that has been termed “mechanotransduction” [13]. This cessation is sensed by a mechanosome within the endothelial cells, leading to the activation of NADPH oxidase to generate ROS and to the activation of nitric oxide (NO) synthase to generate NO [23]. Increased NO causes vasodilatation and ROS provide a signal for revascularization; however, in lung transplantation, overproduction of ROS and NO can cause oxidative injury and/or activation of proteins that drive inflammation and cell death [13].

Furthermore, multiple donor-associated changes can alter the pulmonary vasculature during ischemia, and platelet occlusion of the vascular bed and thrombosis are among the main causes of PGD [24].

### 3.2. Consequences of Ischemia–Reperfusion

**Figure 1:**
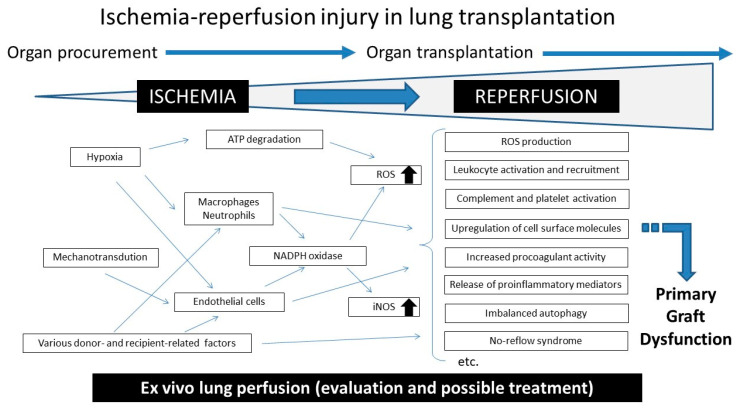
Scheme of ischemia–reperfusion injury in lung transplantation.

In IRI, the process of reperfusion of the graft, not ischemia per se, plays a more important role in causing injury (Figure 1) [3,4]. The consequences of IRI are diverse, including generation of ROS, activation/recruitment of leukocytes, activation of complements and platelets, upregulation of cell surface molecules, increased procoagulant activity, and release of various proinflammatory mediators such as cytokines and damage-associated molecular patterns [25]. These reactions are considerably increased in the lung after reperfusion. 

Increased endothelial permeability is a significant contributor to the development of pulmonary IRI, resulting in the formation of an endothelial gap. Lung IRI induces the migration of leukocytes into the extravascular space, contributing to increased microvascular permeability and increased gaps between endothelial cells. It is also known that IRI is biphasic, involving an acute injury phase characterized by macrophage activation followed by a neutrophil-dependent injury phase [26]. Lung IRI-induced inflammation results in increased apoptosis due to elevated levels of proinflammatory mediators, such as tumor necrosis factor-alpha, interleukin (IL)-1 beta, and IL-8, which contribute to pulmonary dysfunction. Furthermore, lung IRI results in the activation of sterile, innate, and adaptive immune responses by various lung cell types, which are driven by the release of chemokines, cytokines, and other endogenous molecules. Among them, neutrophil graft infiltration and formation of neutrophil extracellular traps, complement system activation, macrophage activation, and toll-like receptor activation are well-known processes [27,28]. 

Autophagy promotes cell survival by allowing the regular degradation and recycling of cellular components; however, an imbalance in endosomal and autophagic pathways upon injury can eventually cause cell death [29]. Therefore, autophagy endangers the lungs during IRI, and inhibition of autophagy might alleviate pulmonary IRI. Suppression of mitochondrial autophagy by 3-methyladenine inhibits the apoptosis of endothelial cells and enhances their proliferation via the mechanistic target of rapamycin pathway [30]. 

## 4. Strategies to Prevent Ischemia–Reperfusion Injury

PGD occurs in approximately 10% of transplant recipients. It is associated with a mortality rate of 42% in the month after transplantation, which is sevenfold higher than that in patients without PGD [31]. Pulmonary IRI after lung transplantation is the main reason for PGD, which is also a major risk factor for the development of chronic lung allograft dysfunction (CLAD) [32,33]. Therefore, various strategies to reduce pulmonary IRI after lung transplantation have been conducted in the clinical setting. 

One of the major challenges in lung transplantation is the shortage of acceptable donor lungs, with a low utilization rate of donated lungs of approximately 20% worldwide [34,35]. To counter this severe donor shortage, marginal donor lungs have been used. Marginal donor lungs are lungs that may be transplantable but do not meet the criteria for ideal donor lungs, such as a ratio of arterial partial pressure of oxygen to fraction of inspired oxygen (PaO_2_/FiO_2_) of > 300 mmHg, absence of infiltration on chest radiographs, clear bronchoscopic findings, and absence of a smoking history [3,36].

The first step in preventing PGD is to eliminate or reduce potentially modifiable risk factors; thus, it is important to understand the risk factors for PGD of both donors and recipients. Second, the greatest efforts have been focused on the method of reperfusion and ventilation of lung grafts in addition to lung preservation. Prolonged ischemic time is associated with a significantly higher risk of PGD; however, surgical and anesthetic considerations are also crucial in PGD prevention [10]. Attempts to reduce the development of PGD have included shortening the ischemic time, controlling reperfusion, using protective ventilation, and using various medications for modulating the IRI pathways [37,38].

### 4.1. Donor and Recipient Factors

Multiple clinical factors related to an increased risk of PGD development have been reported. Among donor-inherent variables, age (>45 or <21 years) and female sex were reported to be clinical risk factors for PGD [39]. Premortem hypoxemia/hypotension and smoking history in the donor, in addition to age and sex, have been reported to be associated with an increased risk of PGD [40]. Furthermore, other donor-acquired risk factors, such as trauma and aspiration, are also reported to be risk factors for the development of PGD. Meanwhile, recipient factors such as body mass index, sex, pulmonary hypertension, and idiopathic pulmonary fibrosis have been reported to be other risk factors for PGD [41]. Factors related to organ procurement and recipient surgery, including ischemic time, single- versus double-lung transplantation, use of cardiopulmonary bypass, and transfusion requirements, have all been implicated in the development of pulmonary IRI and/or PGD. In addition, donor–recipient size mismatch is a major modifiable risk factor for PGD, as oversized allografts have been associated with a decreased risk of postoperative PGD, especially in patients without chronic obstructive pulmonary disease [42]. Furthermore, PGD is a major risk factor for the development of CLAD including bronchiolitis obliterans syndrome [32,33]. 

### 4.2. Procurement and Preservation

Because IRI-induced PGD and donor shortage are more common in lung transplantation than in the transplantation of other organs, the development of a highly effective and reliable organ preservation solution would contribute to improving the function of transplanted organs and to alleviating the shortage of donor organs by enabling the use of marginal donor lungs [3,4]. The Euro–Collins solution has been used in lung transplantation since its first clinical application in renal transplantation in the 1960s. Additionally, the University of Wisconsin solution has also been utilized in lung transplantation. Both solutions are intracellular-type preservation solutions whose higher potassium levels might result in the constriction of the pulmonary arteries [19]. To produce a more reliable preservation solution, many studies have been performed on the regulation of inflammatory cascades contributing to endothelial injury. Extracellular-type solutions with low potassium levels have been developed, and low-potassium dextran preservation solution has been clinically used in many institutions worldwide [43]. In Japan, a novel preservation solution named ET-Kyoto solution, which has a sodium level of 100 mEq/L and a potassium level of 43.5 mEq/L, has been developed and used with favorable outcomes [19,44]. For example, lungs preserved in ET-Kyoto solution have been reported to show satisfactory postoperative function, despite the long preservation time, leading to excellent long-term survival [44]. Although various preservation solutions are currently used in lung transplantation, no multi-institutional randomized controlled studies comparing the different preservation solutions have been performed to date [45]. 

Retrograde flush refers to the administration of a flush solution through the pulmonary veins, with drainage through the pulmonary artery. As an additional retrograde flush has been proven to improve lung preservation compared with an anterograde flush alone, this procedure has been performed in addition to the conventional anterograde flush in many lung transplantation programs. In some cases, a retrograde flush is performed in situ at the donor hospital and a late retrograde flush is performed at the recipient hospital. Recently, a retrograde flush was reported to be more protective than heparin even in uncontrolled lung donations after circulatory death [46].

### 4.3. Protection in Reperfusion and Ventilation

During the last decades, many of the proteins, receptors, mediators, and inflammatory cascades participating in endothelial injury in pulmonary IRI have been targeted by treatment strategies, either with antibodies, inhibitors, or modulators of the inflammatory milieu [47]. One of the other methods for endothelial protection is the progressive reintroduction of blood flow during the initial period of reperfusion, which has been shown to reduce lung injury and to improve the function of the transplanted lung in several experimental settings [3]. In clinical practice, the so-called controlled reperfusion is currently performed in many lung transplantation centers. Furthermore, a protective mode of ventilation after reperfusion is recommended to prevent IRI and ventilator hypercapnia is recommended [48]. An international survey involving 18 countries reported that lung-protective approaches were applied in mechanical ventilation after lung transplantation [49]. Low tidal volumes based on recipient characteristics were also frequently chosen. In contrast, donor characteristics were often not considered and were frequently unknown to the team managing the mechanical ventilation of the recipient after lung transplantation. Multicenter randomized controlled trials are warranted to identify ideal ventilator strategies after lung transplantation [50]. More recently, an international study found that three ventilation parameters (peak inspiratory pressure, PaO_2_/FiO_2_, and dynamic compliance) predict prolonged mechanical ventilation after lung transplantation [51]. Furthermore, venoarterial extracorporeal membrane oxygenation (ECMO) has recently been accepted by many institutions as the primary supportive treatment during lung transplantation [52,53]; however, prolonged use of ECMO after lung transplantation has been reported to yield better results when oxygenation is poor or pulmonary pressures are increased at the end of transplantation. A liberal ECMO prolongation strategy can counteract significant structural damage and prevent severe PGD [54].

### 4.4. Donation after Cardiac Death and Ischemia-Reperfusion Injury

The use of lungs from donation after cardiac death (DCD) donors is one of the options to overcome the problem of organ shortage in lung transplantation. According to data from the International Society of Heart and Lung Transplantation Thoracic Transplant Registry, among patients transplanted between 2003 and 2017 at 22 international centers participating in the DCD Registry, the proportion of DCD lung transplantations performed each year increased from 0.6% in 2003 to 13.5% in 2016 [55]. DCD lung transplantations remain underperformed in the United States. Nevertheless, the survival rate was reportedly similar to that of lung transplantations from donation after brain death (DBD). PGD was reported to be worse in DCD recipients than in DBD recipients on intensive care unit arrival, although subsequent improvements were observed [56]. Unlike DBD lungs, DCD lungs are exposed to warm ischemia in addition to cold ischemia, which should be overcome to control pulmonary IRI [57].

## 5. Current Therapies and Promising Future Possibilities

To date, various experimental studies have been conducted and translated into the clinical setting [58]. One of the most important innovations in the last two decades is the development of ex vivo lung perfusion (EVLP), which has become a routine clinical procedure in large lung transplantation centers in North America and Europe for the assessment of marginal donor lungs. Some of the promising results are detailed below.

### 5.1. Ex Vivo Lung Perfusion

Marginal donor lungs and DCD lungs have begun to be used to resolve the shortage of brain-dead donors [9]. In 2000, Steen et al. successfully performed, for the first time, lung transplantation using DCD lungs after donor lung evaluation using a novel technique called EVLP, which was the starting point for the “Lund protocol” [59]. The Lund group also performed six clinical lung transplantations with marginal donor lungs which were evaluated using EVLP between 2006 and 2007 [60]. Thereafter, a Toronto group also reported the results of a sensational clinical trial that used EVLP to evaluate the function of marginal donor lungs and DCD lungs, which laid the foundation for the “Toronto protocol” [2]. The group performed 372 EVLP procedures between 2008 and 2017, increasing the annual lung transplantation rate by 70% during this period [61]. In 2012, the first-in-human experience using the portable Organ Care System (OCS) lung device for concomitant preservation, assessment, and transport of donor lungs was reported, which was named the “OCS protocol” [62]. Currently, these three types of EVLP have been used for clinical lung transplantation in large lung transplantation centers in North America and Europe, demonstrating that marginal donor lungs could be assessed and treated to achieve outcomes similar to those of donor lungs meeting the standard criteria. Furthermore, the EVLP technology has attracted increasing attention from transplantation centers worldwide [34,63].

**Figure 2:**
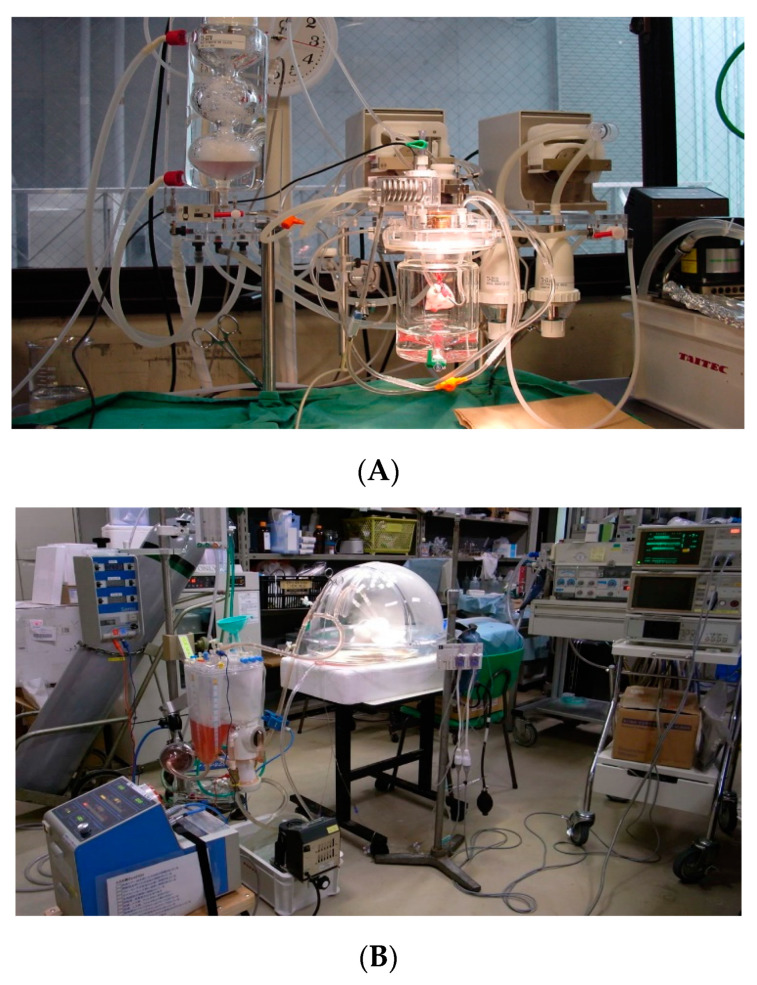
(**A**) Rodent ex vivo lung perfusion (EVLP) model. (**B**) Swine EVLP model.

In addition to clinical application, various experimental studies worldwide have used EVLP to date, thus continuously providing insights into the true meaning of the utilization of this technique [46,64,65,66,67,68]. Preclinical studies have also been performed with human lungs rejected for transplant, lungs from large animals such as swine, and lungs from small animals such as rats and mice (Figure 2) [69]. As a novel strategy of donor lung management, EVLP can keep the donor lungs in a physiologic protective condition and has the potential to increase lung utilization not only by reevaluating but also by treating and repairing the donor lungs before transplantation. 

Owing to similarities to human anatomical and biological features, the porcine EVLP model provides an ideal preclinical platform, and its protocols closely adhere to human protocols [69]. The Toronto group led by Keshavjee and Cypel performed many pivotal studies, starting with the use of extended EVLP to maintain the performance of porcine lungs for 12 h [70]. The Leuven group led by Van Raemdonck also performed studies on strategies such as retrograde flush in DCD donors [46,69]. After the first human lung transplantation using EVLP, the Lund group continued their studies using a porcine EVLP model [46,71]. Furthermore, the Milan group also performed several EVLP studies, such as investigations into the effect of beta-adrenergic agonist infusion during EVLP [72]. Several other groups from European countries such as Germany and France also performed relevant studies using porcine EVLP models [69,73,74].

In a rat EVLP model, pulmonary IRI has been evaluated using physiologic parameters such as pulmonary vascular resistance, shunt ratio, and airway resistance and compliance. Molecular and pathologic analyses have also been performed to investigate the mechanism of pulmonary IRI [19,57,75,76]. Many studies have been performed with a short-term perfusion period in a rat EVLP model. It was demonstrated that EVLP per se elicits an inflammatory response that mimics IRI; however, an anti-inflammatory protective effect was also recently shown in a model of prolonged rat EVLP with a 180 min perfusion period, without the addition of any anti-inflammatory drugs [75]. Some authors have reported a model of 240 min EVLP with the addition of steroids in the perfusate [76], whereas other studies used EVLP models with shorter perfusion periods with or without the addition of anti-inflammatory drugs [57]. The short perfusion time and the application of anti-inflammatory strategies can alter the comprehension of rat EVLP results. The comprehension of these insights is important to properly understand the interplay between IRI and EVLP. Furthermore, by comparing the effects of EVLP and transplantation of human lungs using a transcriptome-wide approach, novel therapeutic targeting associated with inflammation and apoptosis during EVLP might allow for lung repair before implantation and improve the transplantation outcomes [77].

### 5.2. Novel Strategies for Endothelial Protection

Although autophagy seems to be an important mechanism for the occurrence of apoptosis, studies on targeting autophagy have been scarce and the role of autophagy in pulmonary IRI remains ambiguous. More studies are clearly needed to elucidate whether or not the inhibition of autophagy can provide an endothelium-preserving effect. Another possibility is to stabilize the endothelial glycocalyx against degradation by pulmonary IRI [78]. This layer is the prominent interface between the bloodstream and its constituents and the vascular endothelium. Preventing the initiation of the damaging effects of IRI, such as by preserving the glycocalyx, might be more important than treating the endothelium that is already damaged by IRI. Other options, such as targeting P-selectin and angiotensin-converting enzymes, might also contribute to the amelioration of pulmonary IRI [79,80]. Some of these strategies have been proven to be promising in an experimental setting; however, few of them have been transferred into clinical application. Furthermore, these strategies could be combined with EVLP.

### 5.3. Surfactants

It is known that surfactant proteins are decreased during ischemia, which leads to alveolar wall damage [81]. Surfactant proteins play important roles in maintaining the microstructural integrity of the lungs by providing low surface tension at the air–liquid interface and by preventing alveolar collapse. Therefore, surfactants have been administered to patients with severe PGD after lung transplantation and to donor lungs [82,83,84]. However, despite the various experimental and clinical studies, the use of surfactants in clinical lung transplantation is still rare owing to their high cost [84,85,86]. Furthermore, surfactant inhalation has some adverse effects, such as transient desaturation during bronchoscopy for instillation [83]. Despite these hurdles, several studies have focused on the importance of pulmonary surfactants [87,88,89]. In the future, randomized controlled studies in a larger cohort of patients might be required to further elucidate the optimal dosing, timing of treatment, and routes of delivery.

### 5.4. Inhaled Beta-2 Adrenoreceptor Agonists

Inhalation offers a lung-specific route for drug delivery, and many inhaled drugs for respiratory diseases are available. Beta-2 adrenoreceptor agonists are one of the key drugs for the treatment of asthma and chronic obstructive pulmonary disease. In humans as well as in animals, beta-2 adrenoreceptor agonists intravenously injected or aerosolized and administered through the airway were reported to have protective effects against a variety of pulmonary conditions, such as acute respiratory distress syndrome and pulmonary edema after lung resection [57,72,90]. Beta-2 adrenoreceptors are distributed in alveolar cells, airway epithelium, airway smooth muscle, and pulmonary vessels in humans, which suggests that inhaled beta-2 adrenoreceptor agonists might act on various pulmonary tissues, relaxing the airway and vessels [91,92]. Furthermore, the mechanism by which inhalation provides protection can be inferred from the elevation of cyclic adenosine monophosphate (cAMP) levels [57,92]. Inhaled beta-2 adrenoreceptor agonists were also reported to have protective effects against IRI both in small and large animal models [57,92,93,94]. The mechanism by which the inhalation of beta-2 adrenoreceptor agonists provides protection can be inferred from the maintenance of the cAMP and adenine nucleotide levels, inactivation of inflammatory cells, and reduction of cytokine production.

### 5.5. Therapeutic Gases

Several therapeutic gases are known to have the potential in preventing and treating lung IRI after lung transplantation. First, inhaled NO, an endogenously-derived molecule synthesized by NO synthase, has been used in many lung transplantation programs, although several clinical studies failed to show positive results [3]. Lung transplantation causes pulmonary vascular dysfunction via the upregulation of inducible NO expression, and inhibition of inducible NO synthase reverses post-transplantation pulmonary vascular dysfunction [95]. This supports the importance of controlling NO levels in lung transplantation. However, inhaled NO did not significantly decrease the incidence of lung IRI in a double-blinded, placebo-controlled randomized trial [96]. 

Second, low-concentration carbon monoxide (CO), which is a well-known toxic gas causing lethal poisoning, has been proven to be efficacious in several animal models as well as in clinical settings involving IRI and organ transplantation [97]. CO, an endogenously produced byproduct of heme catalysis, has various functions, including anti-inflammatory effects through the upregulation of potent anti-inflammatory cytokines and antiapoptotic effects via the upregulation of hypoxia-inducible factor (HIF)-1-alpha. Low-dose CO inhalation into transplanted lungs has been reported to have beneficial effects in large animal models and in rodent models. Further, CO could protect against IRI through the induction of HIF-1alpha, the degradation of which has been shown to reduce lung edema and inflammation [98]. Before clinical application, more studies are needed to clarify the role of therapeutic CO in the future.

Third, since the first report by Ohsawa et al., molecular hydrogen has been used as a therapeutic antioxidant in various medical fields [99]. The antioxidative, anti-inflammatory, and antiapoptotic effects of hydrogen therapy have been demonstrated in various models, such as multiple organ dysfunction syndrome and organ transplantation [100]. Hydrogen gas also has potential as a new therapeutic strategy against lung IRI owing to its function as a radical scavenger and its capability to induce substances against antioxidant stress through Nrf2 signaling [101]. In addition, hydrogen preconditioning during EVLP was also reported to improve the quality of lung grafts in rodents [76,102]. It was reported that the combination therapy with NO and hydrogen provided enhanced therapeutic efficacy for pulmonary IRI in a murine model [103]. As hydrogen gas has inflammable and explosive properties, it is unsuitable for transporting organs. Currently, hydrogen-rich solutions (i.e., dissolution of molecular hydrogen in organ preservation solutions) have also been introduced into clinical practice and were reported to alleviate pulmonary IRI in rat and beagle models [104,105,106].

### 5.6. Fibrinolytic Treatment

The pulmonary endothelium is the first site of IRI. Furthermore, multiple donor-associated changes can alter the status of the pulmonary vasculature during ischemia, such as platelet occlusion of the vascular bed and thrombosis. Thrombosis of grafted lungs is inevitably associated with high resistance and inadequate perfusion. Heparin is often used when procuring donor lungs. Although heparin can prevent new fibrin formation, it does not have a lytic effect on pre-existing donor thrombi. Therefore, fibrinolytic treatment of lung grafts using plasminogen activators, such as urokinase and tissue plasminogen activator, has been utilized for the management of donor lungs [107,108,109]. Recently, plasmin administration during EVLP has been shown to dissolve thrombi in the lungs, resulting in reconditioning of the lungs by alleviating pulmonary IRI [110,111]. The success of fibrinolytic treatment seems crucial in DCD donors when timely heparin use might not be possible [112]. The use of fibrinolytic treatment has become a clinical standard in some EVLP centers.

### 5.7. Mesenchymal Stem Cells

The application of mesenchymal stem cells for IRI has been investigated in animal models, showing anti-inflammatory and anti-apoptotic effects, particularly against damage to various organs [113]. Infusion of mesenchymal stem cells has been shown to protect lung transplants from IRI in mice [114]. Mesenchymal stem cells may be an attractive therapeutic strategy owing to their paracrine, immunomodulating, and tissue remodeling properties [115]. Mesenchymal stem cell-derived extracellular vesicles also reportedly have the potential to attenuate lung inflammation in lung IRI [116,117,118,119]. In addition, multilineage-differentiating stress-enduring cells, which are stress-tolerant and nontumorigenic endogenous pluripotent-like stem cells, have been shown to have potential in alleviating pulmonary IRI [120]. Preclinical studies have demonstrated promising results of using mesenchymal stem cells for the treatment of diverse lung disorders including emphysema, bronchopulmonary dysplasia, fibrosis, and ARDS [121]. Mesenchymal stem cell therapy during EVLP has also been shown to ameliorate pulmonary IRI in a pig lung transplantation model [122]. However, before clinical application, the potential of mesenchymal stem cells for spontaneous malignant transformation, depending on the cell preparation process, needs to be considered [123]. A phase 2a safety trial on the use of allogeneic mesenchymal stem cells for the treatment of moderate to severe ARDS was published in 2019, reporting an acceptable safety profile [124]. These promising results are generalizable to the lung transplant population and provide a basis for clinical trials evaluating the efficacy of mesenchymal stem cells in the treatment of pulmonary IRI in lung transplantation [125,126]. Recently, several mesenchymal stem cell therapies for chronic lung allograft dysfunction have been reported. Although long-term results are still awaited, intravenous administration of mesenchymal stem cells is considered feasible and safe in patients with CLAD [127,128].

## 6. Conclusions

Various strategies have been attempted to reduce IRI after lung transplantation, both from the experimental and clinical aspects. Although the pathophysiology of lung IRI remains incompletely understood, maintenance of the integrity of the pulmonary vascular endothelium is pivotal. Recently, EVLP has been clinically introduced worldwide and new therapeutic modalities are awaiting clinical application. Lung IRI consists of complex inflammatory reactions. Therefore, more studies on controlling pulmonary IRI are required to improve the outcomes of lung transplantation.

## Data Availability

Not applicable.
